# Draft Genome Sequences of *Pseudomonas aeruginosa* B3 Strains Isolated from a Cystic Fibrosis Patient Undergoing Antibiotic Chemotherapy

**DOI:** 10.1128/genomeA.00804-13

**Published:** 2013-10-17

**Authors:** Rasmus Lykke Marvig, Nicholas Jochumsen, Helle Krogh Johansen, Niels Høiby, Søren Molin, Morten O. Sommer, Lars Jelsbak, Anders Folkesson

**Affiliations:** Department of Systems Biology, Technical University of Denmark, Lyngby, Denmarka; Department of Clinical Microbiology, Rigshospitalet, Copenhagen, Denmarkb

## Abstract

*Pseudomonas aeruginosa* frequently establishes chronic infections in the airways of patients suffering from cystic fibrosis (CF). Here, we report the draft genome sequences of four *P. aeruginosa* B3 strains isolated from a chronically infected CF patient undergoing antibiotic chemotherapy.

## GENOME ANNOUNCEMENT

Cystic fibrosis (CF) patients are subjected to intensive antibiotic therapy, and it is evident that this crucially affects the process of bacterial adaptation to the CF lung environment (1, 2). To further investigate the effects of antimicrobial chemotherapy on the adaptation of *Pseudomonas aeruginosa* to the CF lung, we sequenced the genomes of four strains of the *P. aeruginosa* B3 clone type isolated from consecutive sputum samples from a patient with long-term chronic *P. aeruginosa* infection undergoing 3 months of combination chemotherapy with oral ciprofloxacin and inhaled colistin due to recurrent isolation of *P. aeruginosa*. The sputum samples were collected and the strains were isolated as part of a longitudinal study where the first and subsequent samples were stored from the beginning of 2005 until July 2009 (1). The first sample was collected before chemotherapy was initiated and is represented by *P. aeruginosa* B3-1811, a mucoid isolate that was determined at the clinic to be colistin and ciprofloxacin sensitive. A sputum sample collected 2 months after the initiation of therapy contained *P. aeruginosa* with two different bacterial colony morphologies: a mucoid and colistin-sensitive strain (*P. aeruginosa* B3-208) and a nonmucoid and colistin-resistant strain (*P. aeruginosa* B3-20M). The last strain (*P. aeruginosa* B3-CFI), also colistin resistant, was isolated from a sputum sample obtained at the end of the 3-month treatment regime. Genomic DNA from the B3-1811, B3-208, B3-20M, and B3-CFI strains was prepared and sequenced as described previously (3) to generate 3,077,857, 2,445,795, 3,026,144, and 3,881,715 75-bp reads, respectively.

Using Velvet version 1.0.16, the reads were assembled into the draft genomes of strain B3-1811, with 6,657,892 bp, 285 contigs, and an N_50_ of 82,268 bp; strain B3-208, with 6,701,506 bp, 344 contigs, and an N_50_ of 66,611 bp; strain B3-20M, with 6,704,349 bp, 278 contigs, and an N_50_ of 77,595 bp; and strain B3-CFI, with 6,727,794 bp, 252 contigs, and an N_50_ of 87,472 bp. The optimal assembly settings were estimated using VelvetOptimiser (the Victorian Bioinformatics Consortium [VBC], Monash University; see http://www.vicbioinformatics.com/software.velvetoptimiser.shtml), with only contigs of ≥500 bp retained, and the assembly was assisted by initial mapping of the reads against the reference genome sequences of *P. aeruginosa* PAO1 (4) using the Velvet Columbus module.

A comparison of the genomes using MUMmer3 (5) revealed the four isolates to be closely related, sharing at least 99.6% of their genomic contents. The sequences of the four strains reported here will help to elucidate the genetic diversity that exists within clonal populations of *P. aeruginosa* sampled from a single CF patient and how the genetic composition can change quickly due to new selection pressures, such as antibiotic therapy.

### Nucleotide sequence accession numbers.

These genome sequences have been deposited in EMBL under the following accession no. (BioProject no.): CBMP010000001 to CBMP010000341 (PRJEB4309) for strain B3-1811, CBMT010000001 to CBMT010000418 (PRJEB4310) for strain B3-208, CBMU010000001 to CBMU010000338 (PRJEB4311) for strain B3-20M, and CBMS010000001 to CBMS010000314 (PRJEB4312) for strain B3-CFI.
